# Major centers of motion in the large ribosomal RNAs

**DOI:** 10.1093/nar/gkv289

**Published:** 2015-04-13

**Authors:** Maxim Paci, George E. Fox

**Affiliations:** Department of Biology and Biochemistry, University of Houston, 4800 Cullen Blvd. Houston, TX 77204-5001, USA

## Abstract

Major centers of motion in the rRNAs of *Thermus thermophilus are* identified by alignment of crystal structures of EF-G bound and EF-G unbound ribosomal subunits. Small rigid helices upstream of these ‘pivots’ are aligned, thereby decoupling their motion from global rearrangements. Of the 21 pivots found, six are observed in the large subunit rRNA and 15 in the small subunit rRNA. Although the magnitudes of motion differ, with only minor exceptions equivalent pivots are seen in comparisons of *Escherichia coli* structures and one *Saccharomyces cerevisiae* structure pair. The pivoting positions are typically associated with structurally weak motifs such as non-canonical, primarily U-G pairs, bulge loops and three-way junctions. Each pivot is typically in direct physical contact with at least one other in the set and often several others. Moving helixes include rRNA segments in contact with the tRNA, intersubunit bridges and helices 28, 32 and 34 of the small subunit. These helices are envisioned to form a network. EF-G rearrangement would then provide directional control of this network propagating motion from the tRNA to the intersubunit bridges to the head swivel or along the same path backward.

## INTRODUCTION

The ribosome is a dynamic molecular machine that is responsible for coded protein synthesis. It is comprised of two subunits, each of which consists of RNA and protein. During protein synthesis the ribosome passes through four functional phases: initiation, elonga­tion, termination and recycling while transitioning between rotated and unrotated states (1). In Bacteria, the major co-factors that facilitate the process are the elongation factors EF-Tu, EF-G, IF-2 and the release factor RF-3. The ribosome is a Brownian motor where the conformational changes are actually an inherent property of the ribosome itself (2). Thus, intersubunit rotation can occur spontaneously and reversibly without guanosine triphosphate (GTP) hydrolysis (3–5). EF-G likely serves to coordinate and hasten the process by cycles of conformational rigidity and relaxation before and after GTP binding (6,7).

During translation, transfer RNAs carrying amino acids previously attached by the aminoacyl tRNA synthetases enter the ribosome in response to codons in the mRNA. An incoming tRNA is initially accommodated into the A-site, and then moved to the P site following peptide bond formation and from there to the E-site where it will exit the ribosome. Initial crystal structures revealed a hinge-like region or pivot point in the tRNA (8,9). The motions of tRNA during the various stages of translation including accommodation are largely associated with reorientations of this hinge (10). Similarly, pivot points can serve as fulcrums that facilitate helix reorientation in the large RNAs (11).

In order to understand ribosome dynamics, a number of investigators have determined high-resolution structures before and after the EF-G associated GTPase cleavage. These events have now been characterized at atomic resolution in several crystal structures, which show the trapped EF-G-ribosome complex (7,12,13). Multiple investigators have focused attention on one or more motions. These include the flexibility of the L1 stalk three-way junction (14,15), the putative origins of head and body movement as seen in high-resolution structures and in cryo-EM studies (16,17), molecular dynamics studies (18), and extensive all atom-simulations that identify atomic positions that show minimal movement during large structural movements in the ribosome. Most recently, a detailed investigation of the origins of 30S subunit head movement across multiple crystal structures was provided (19). All of these studies have computationally analyzed motions within ribosome structures at different levels, using all-atom simulations or variation of atomic positions across different structures and many have identified specific locations in ribosomal RNA where movement is likely to originate. In particular, Sanbonmatsu *et al*. (20–23) have attempted, on multiple occasions, to identify the direction and nature of movement within the ribosome.

Herein, we tabulate likely pivoting positions in the large rRNAs of *Thermus thermophilus* and determine the extent to which equivalent pivot points are found in the large rRNAs of *Escherichia coli* and *Saccharomyces cerevisiae*. This includes instances of minor pivots that have not been explicitly pointed out previously. Knowledge of the location of pivots in the rRNAs will enhance our understanding of the cascades of motion undoubtedly associated with translation and provide insight into when this important aspect of the modern machinery came into existence in the context of ribosome evolutionary history (24).

## MATERIALS AND METHODS

**Figure 1.**
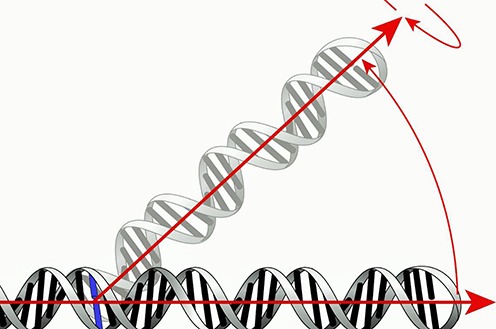
Illustration of how pivot points are identified. A rigid stem sequence from the two structures being compared is superimposed. The nucleotide mismatch or motif where one strand's increasing deviation from the next originates is the pivot point. The loop sequence that completes the pivot structure is shown in gray. The arrows show directionality toward the loop of the measured helices and the freedom of these helices to move in 3D space about the pivoting position. The arrows diverge from one another at the pivot point.

Pivot points were identified through a two-step process. Structure-based global superposition was performed on the small subunit (SSU) and large subunit (LSU) rRNAs before and after EF-G binding. Particularly mobile A′-form helices were identified and additional alignments were performed to identify positions, which would yield the largest motion. The identified pivoting helices were then subdivided into three segments as indicated in Figure 1. These are: (i) a rigid stem sequence, which is subject to local sequence alignment, (ii) the nucleotide mismatch or motif, which initiates one strand's increasing deviation from the next—‘the pivot point’—and (iii) the loop sequence, which completes the pivot structure. By aligning rigid stem sequences which show no significant motion as the result of EF-G binding one avoids problems with changes in atomic positions that would occur if the alignment were done on the hinge itself. For example, in the case of helix h28 a short stem sequence was aligned upstream of the possible pivots in h28. The resulting change is seen in ‘the final loop’ sequence, which in this case ends in helix 34 and RNAs extending from it.

The motion is quantified in angstroms as the difference in the distance from the nucleotide backbone furthest away from the pivot before and after EF-G binding. The approach establishes the presence of a pivot point and provides a good approximation of where it is located. Although the approach is generally robust, it may fail if there is no rigid stem sequence available for alignment or if the range of motion is smaller than the crystal structure resolution. Another potential problem is movement from crystal conflicts or areas with large B-factors. It is not always clear if a high B-factor is the product of inherent ‘flexibility’ of the RNA or that the observed flexibility is simply an artifact of a disorganized crystal structure. However, likely pivot points described here to the extent they were previously known agree well with earlier literature reports. In addition, we have sampled a series of crystal structures to address this.

Two series of structural comparisons were carried out using the PyMOL Molecular Graphics System, Version 1.5.0.4 Schrödinger, LLC. (http://www.pymol.org). The first comparison set contrasts large subunit structures that are EF-G bound and unbound. The second comparison set describes the difference in small subunits. Full 70S structures were not compared as the relevant bridging contacts between the subunits are known and discussed at length in the literature. This approach decouples global motions available to the 70S from the EF-G-dependent motions of interest here.

All structures were obtained from the PDB (25), (http://www.rcsb.org). Structures 2J01 and 2J02, now included in 4V51 (26), were used as the reference non-rotated state in *T. thermophilus*. A global alignment of these two structures with earlier published non-rotated structures 2WDI and 2WDG (27) now listed as 4V5C was undertaken. The RMSD was 0.432 for the 16S rRNA and 0.345 for the 23S rRNA after removal of all non-rRNA structures. These RMSD values provide an indicator of the variation that must be exceeded to indicate meaningful differences. Structures 2J01 and 2J02 were next compared against structure pairs 4JUW, 4JUX in entry 4V9H (12), which purport to show the ribosome in an intermediate state of rotation. In this case, the RMSD values were 1.951 for the 16S rRNA and 0.911 for the 23S rRNA far exceeding the cutoff values as did all the other comparisons undertaken. This magnitude of difference was seen across all EF-G bound versus unbound structures. More importantly however, local alignments, unperturbed by the global 70S state, showed a large difference in motion in comparison to the standard structures.

A structure thought to represent a fully ratcheted state was also compared, using PDB files 2WRI, 2WRJ now listed as 4V5F (28). To assess the extent of conservation of pivot locations additional comparisons were undertaken using *E. coli* and *S. cerevisiae* structures. The standard *E. coli* structures used for these comparisons were 3R8T and 4GD2, which are now assigned to PDB entry 4V9D (29). These were compared against structures 4KIX, 4KIY in entry 4V9O (7) and 3R8S, 4GD1 now entry 4V9D (29) thought to represent the classical, intermediate and final ratcheted states of the *E. coli* ribosome, respectively. Finally, the very recent structures, 3J77 and 3J78 (30), showing the classical and fully rotated states of the yeast ribosome were compared in order to ascertain whether pivots in the yeast RNA are likely present at similar locations as in the Bacteria. These 6-Å resolution cryo-EM structures were previously subject to real-space refinement against a 3-Å crystal structure (31). The accuracy of the fit was assessed using a Fourier shell correlation (30). The resolution of these structures is therefore thought sufficient for meaningful comparison.

The stems were aligned using the ‘align’ command in PyMOL, which forces a minimal distance between all atoms of the stem sequence. Though the function does ignore a fraction of compared atoms to produce a visual best fit it is suitable for the purposes of highlighting the existence of large mobile elements. Measurements made using this method are relative since the choice of aligned sequences has an effect on the magnitude of the pivot. Nevertheless, this method accurately highlights elements in the ribosome that are known for their mobility and functionality. Single Watson–Crick matches were found suitable for alignment sequences as they would yield the superposition of at least 30 atoms—enough to generate reproducible directionality. The magnitude of motion was measured by the displacement of a nucleotide in the final loop of the helix. Finally, to the extent possible, nucleotide positions were labelled according to the usual *E. coli* rRNA numbering.

## RESULTS

**Figure 2.**
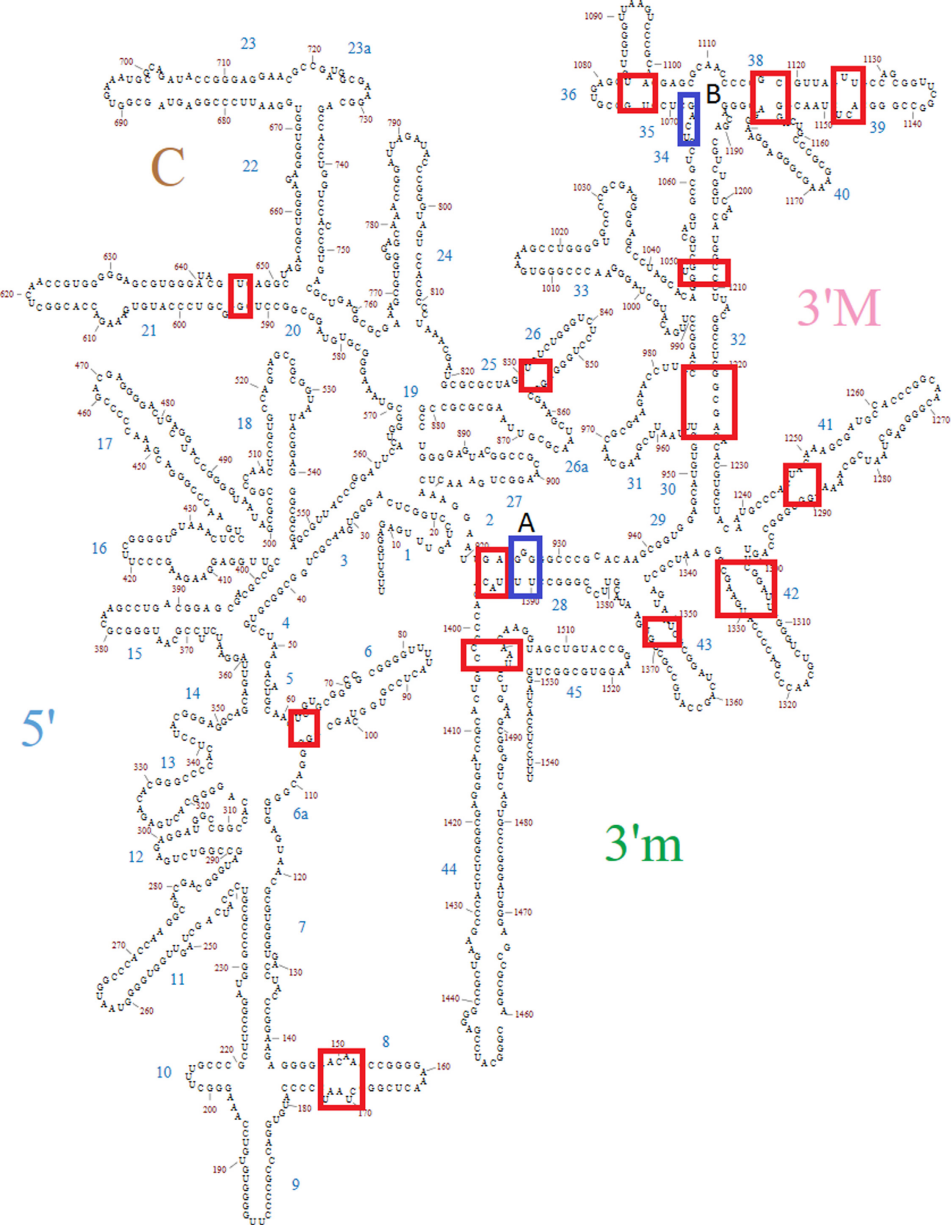
The location of the pivots identified in *T. thermophilus* 16S rRNA is indicated in rectangles on a secondary structure diagram. The blocks indicating the pivots are of varying size depending on how well determined the pivot location is. The blocks labeled A and B are pivots suggested by Mohan *et al*. (19) as discussed in the text. Block A represents a possiblealternative pivot in helix 28. THe pivot represented by Block B can't be evaluated by the approach described herein.

**Figure 3.**
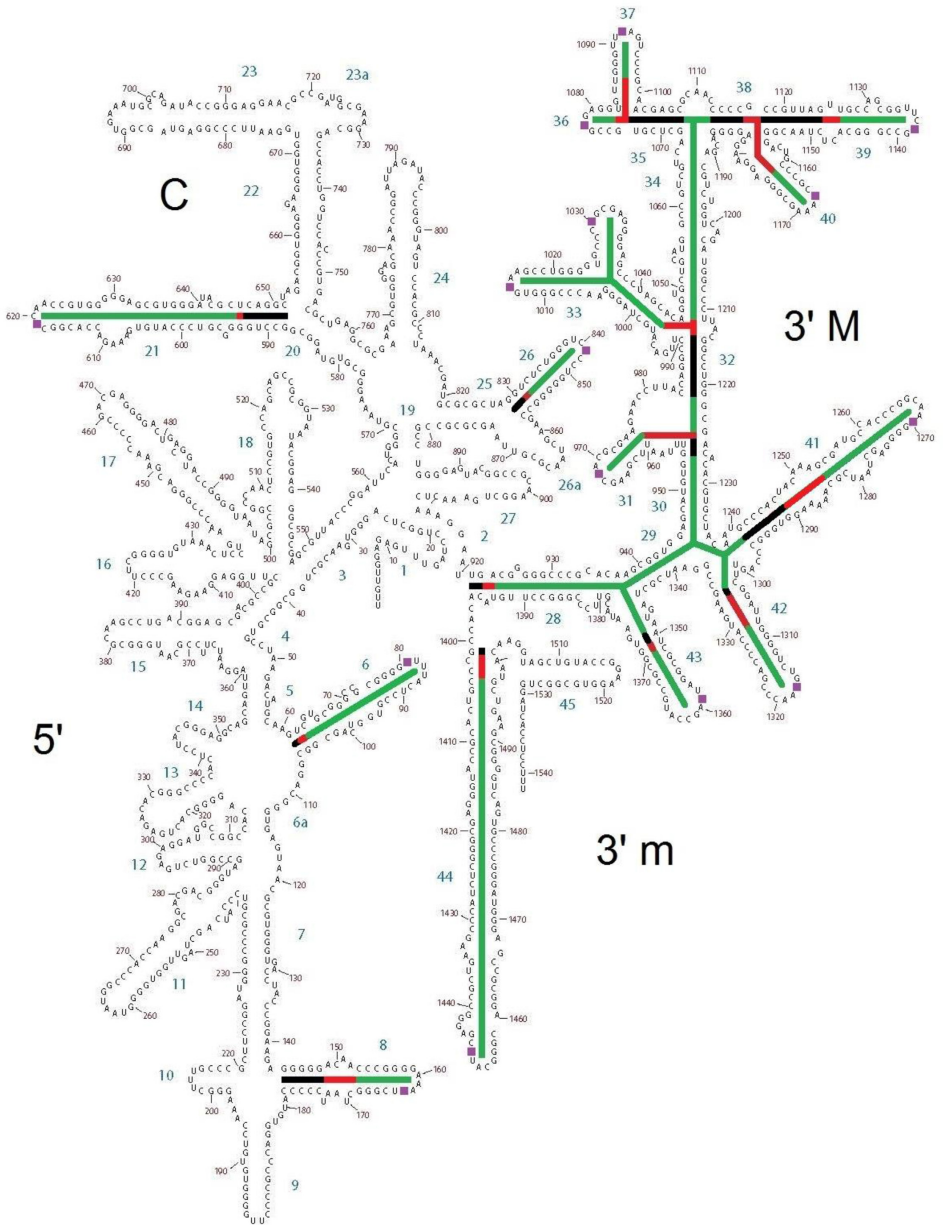
Secondary structure of *T. thermophilus* 16S rRNA highlighting the details of each pivoting element. The stem sequences that were superimposed are highlighted in black. Pivoting elements are shown in red and final stems are shown in green. Helices 29 and 34 are considered to be final helices by our definition of a pivot, even though they are internal. They are therefore color coded in green as are the external final helices. The figure thus highlights the connectivity between pivoting helices 28, 32 and the remainder of the SSU head domain.

**Figure 4.**
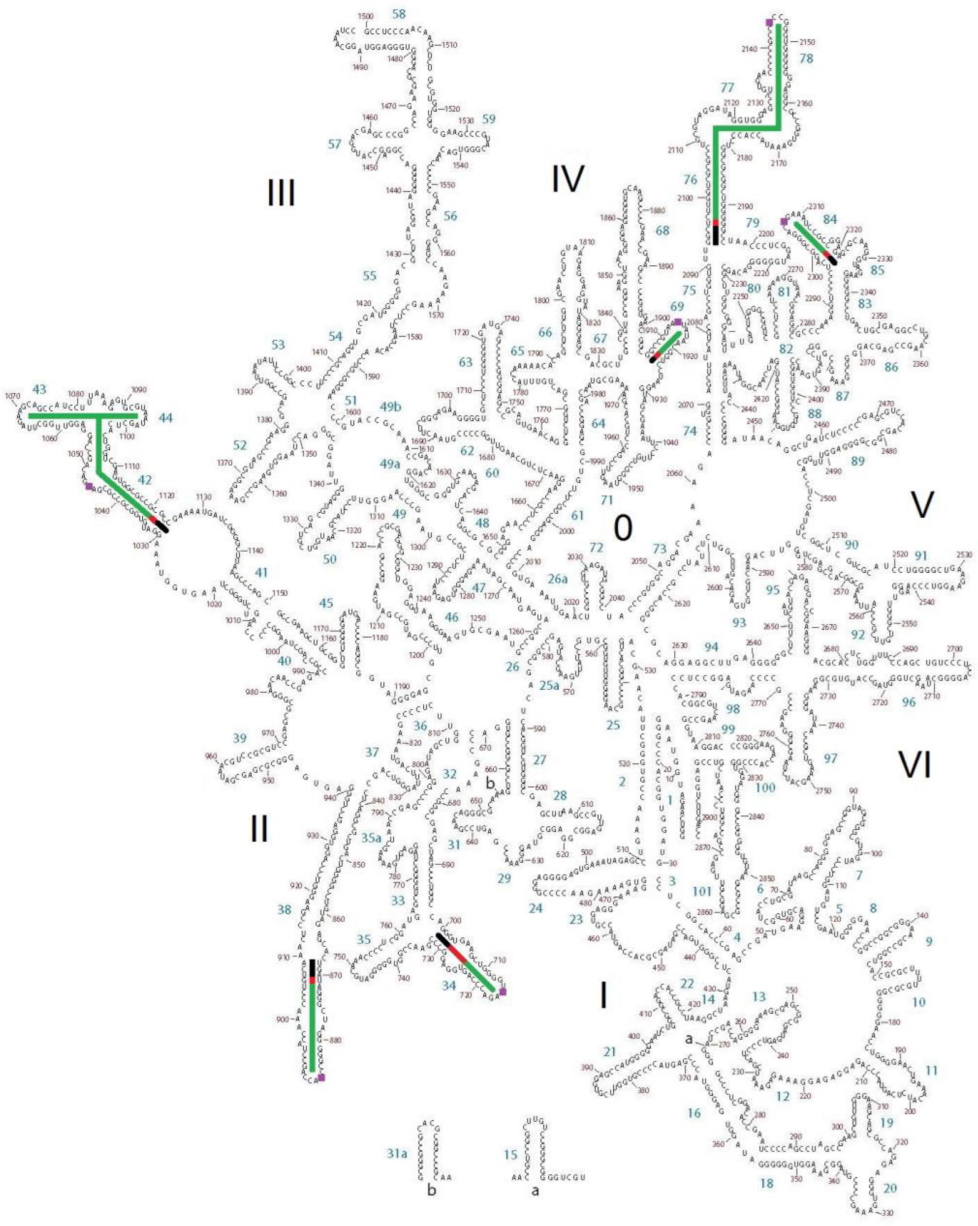
Secondary structure map of *T. thermophilus* 23S rRNA highlighting pivoting elements. Aligned stem sequences are highlighted in black. Pivoting elements are shown in red and final stems are shown in green as in Figure 3.

Initially, elongation factor G (EF-G) unbound ribosomes (26) from *T. thermophilus* were compared with EF-G bound structures in various states (12,28). These comparisons revealed 21 hinge-like regions in the 16S and 23S rRNAs, which likely act to accommodate the forward translation process. Of these, many were not previously explicitly described. The newly discovered pivot points are found primarily in the small subunit in helices h6-the spur, h8, h21, h26 as well as in the majority of the helices in the 3′ major domain (h31, h32, h33, h36, h37, h39, h40, h41, h42 and h43). The location of these pivots is shown in the context of the *T. thermophilus* 16S rRNA secondary structure (Figure 2). Pivots found in the 23S rRNA are in helices H34, H38, H42, H69, H76 and H84. Their location is shown on Supplementary Figure S1 utilizing the secondary structure model that was recently derived from tertiary structure (32,33). More detailed displays that also highlight the stems that were superimposed and final stems are shown in Figures 3 and 4. Subsequently, 12 additional comparisons were undertaken for *E. coli* (7,29) and *S. cerevisiae* (30) ribosomes. Equivalent pivots were typically found, thereby demonstrating their conservation. It should be noted, however, that intersubunit rotation may not always be correlated with head rotation or L1 stalk movement.

The precise location of the pivots was frequently, but not always, the same in all three organisms. The locations are summarized in Table 1. Secondary structure diagrams showing the location of the *E. coli* and *S. cerevisiae* pivots in the same format as Figure 3 are provided as Supplementary Figures S2–S5. In addition to identifying the likely location of each pivot, the structure alignments provide insight into the magnitude of motion associated with each position. These measurements are summarized in Table 2. Full details for each individual crystal comparison are provided as Supplementary Tables S1–S12.

**Table 1.** Location of pivoting positions in large and subunit rRNAs

LSU helix	*T. thermophilus*	*E. coli*	*S. cerevisiae*–*E. coli* #s	*S. cerevisiae*
34	703-bulge	703 U-G	701 G-U	832 G-U
38	871 U-G	870 U-G	839 U-G	986 U-G
42	1032 U-G & bulge A	1032 U-G & bulge A	1033 bulge U	1208 bulge U
69	1907 G-U	1907 G-U	1907 G-U	2250 G-U
76	2096 U-G	2099 U-G	2095 G-U	2437 G-U
84	2298 bulge	2298 bulge	2298 bulge	2667 bulge
SSU helix				
6	62 U-G	62 U-G	62 bulge	58 bulge
8	149 bulge	145 G-G	148 bulge	143 bulge
21	593 G-U	589 U-G	No analog	No analog
21es6c	No analog	No analog	Not defined	746 bulge
26	831 U-G	832 G-U	832 bulge	1042 bulge
28	1394 bulge A	1394 bulge A	1394 bulge A	1631 bulge A
31	955 3wj	955 3wj	955 3wj	1181 3wj
32	1212 bulge	1212-bulge	1212 bulge	1444 bulge
33a/b		1042-A Bulge	1040 Bulge	1225 bulge
36/37	1074 3wj	1074 3wj	1071 G-G 3wj	1291 G-G 3wj
39	1125 bulge	1125 bulge	1125 (Est) bulge	1346 bulge
40	1156 3wj	1156 3wj	1155 (Est) 3wj	1387 3wj
41	1247 bulge	1242 G-U	1247 (Est) bulge	1481 bulge
42	1304 bulge	1304-bulge	1304 bulge	1541 bulge
43	1351 U-G	1351 U-G	1351 G-U	G1588 G-U
44	1402 bulge	1402 bulge	1402 bulge	1639 bulge

All *T. thermophilus* position numbers are given as *E. coli* equivalents. To the extent possible, *E. coli* position numbers are also provided for *S. cerevisiae* (column 3). In addition, the actual *S. cerevisiae* positions are given (column 4). In some cases, the *E. coli* numbers are approximations and entered as estimates (Est). In instances where specific base-base interactions are identified the base type is followed by the position number. In the case of LSU helix 38 that region of the 16S rRNA was not well resolved in all crystal structures. In many cases, the pivot site is associated with a bulge and is indicated as such in the chart with the start position indicated.

**Table 2.** Magnitude in angstroms of the motion of the final loop residues as a result of superposition of stem sequences

LSU helix	*T. thermophilus* (Å)	*E. coli* (Å)	*S. cerevisiae* (Å)
34	4.0	2.7	3.4
38	8.3	Incomplete	5.2
42	3.1	7.9	4.3
69a	4.7	2.8	2.9
76	18.8	Incomplete	8.6
84	4.1	1.4	2.3
SSU helix	*T. thermophilus*	*E. coli*	*S. cerevisiae*
6	12.4	7.30	3.4
8	3.5	3.50	1.8
21	2.3	3.80	11.9
26	3.1	2.90	4.8
31	0.70	0.60	3.3
33a	NA	5.80	3.5
33b	NA	0.90	3.8
36	1.2	0.50	4.2
37	1	0.80	4.6
39	5.2	1.50	7.1
40	3.7	2.2	2.9
41	0.50	0.90	1.7
42	1.60	3.40	2.2
43	0.60	3.40	3.3
44	5.4	3.50	18.1

Locations of the pivoting positions are listed in Table 1. Instances where the structure is not adequately resolved are listed as incomplete.

Further examination of these measurements revealed a possible network of motions resulting from the EFG domain open state binding to the ribosome. In each case a pivot was in direct contact with at least one, often several others. It is unknown, however, whether these elements all function simultaneously, as a cascade or as separate elements or groups of elements. Intersubunit rotation may not always be correlated with head rotation or L1 stalk movement for example.

Alignment along the stem of h28, which has previously been associated with the head swivel (23,34), revealed a large-scale motion in essentially every helix of the 3′ major domain. If one first aligns helix h28, the amount of repositioning associated with helices h31, h33b, h36, h41, h42 and h43 is far greater than what is observed when the stems of these helices are separately aligned (Table 3 and Supplementary Tables S13 and S14). Likewise, initial alignment along the stem of the more external h32 showed large-scale motions in helices h36, h37, h39 and h40 compared to individually aligned motions. Helices h28 and h32 are therefore likely to be primary pivots whose motions control separate sets of the more external helices.

**Table 3.** Motions in angstroms at the final nucleotides of the 5′ major domain helices in *T. thermophilus* as a result of pivoting at helices h28 and h32

SSU helix	Helix 28 (Å)	Helix 32 (Å)	Individual (Å)
31	3.40	NA	0.70
33a	9.3	4.70	NA
33b	8	0.20	NA
36	3.6	3.1	1.2
37	1.7	2.3	1
39	4.8	6.3	5.2
40	2.1	4.9	3.7
41	6.4	NA	0.50
42	6.30	NA	1.60
43	4.20	NA	0.60

Alignment at the stem of helix 28 yields changes listed in the leftmost column, alignment at the stem in helix 32 yields the middle column and alignments at the external helixes’ individual stem yields the column on the right.

With regard to the location of the likely pivot points, a general trend was observed. In almost every case, pivots are associated with a weak single base pair mismatch or bulge near major helical junctions. A range of possible orientations is then made available for the rRNA regions that likely facilitate functionality. When the pivot is associated with a bulge it is typically was not possible to assign it to a particular residue. Of the 21 pivots observed in *T. thermophilus*, seven are likely associated with a G-U wobble base pair that can introduce helical irregularities (35).

## DISCUSSION

Structural studies have shown that the 3′ major domain of the small subunit is particularly mobile (18) and functionally important with respect to helix h34 (23,36–38). Specifically, h34 has a binding site for spectinomycin (39,40) and is also known to be important for the decoding process where it has been proposed to participate directly in the termination of translation at UGA stop codons (41,42). The present analysis extends our understanding of the head swivel by showing that motion at h28 (Table 3) influences the motions of a full network of flexible 3′ major domain helices including h31, h33b, h36, h41, h42 and h43. In addition, h32 is also likely to be a key controlling element as it independently influences the motions in helices h37, h39 and h40 (Table 2). These findings regarding the 3′ domain of the small subunit are consistent with recent work in which it is argued that straightening of kinked helix h28 at a pivot near position 926 in combination with a pivot in a three-way junction in h34 produces the head swivel motion through rotation about an imaginary axis (19). These putative pivots are indicated as Block A and Block B on Figure 2.

The two potential pivots in h28 may work together or separately. Our measurements (Supplementary Table S15) reveal that the pivot at 1394 is associated with considerably larger motion than the possible alternative pivot (19) associated with the bulge at 926. The latter primarily influences helices h33a and h39 by measurements undertaken here. We are unable to verify the pivot that has been suggested in helix h34 (19) because our method requires the presence of a reliable upstream stem sequence, which is not available in this case. We argue that the head domain does not function as a rigid structure as others have suggested (19–21). Instead a set of connected flexible elements together makes up the rotation about the imaginary axis (19) of the head swivel. We further argue that pivots in h28 and h32 exert an effect on smaller pivots closer to the exterior of the head domain, moving surface portions of the rRNAs in a manner that may facilitate function and directly relate rRNA motion to protein periphery. These motions have been generally discounted as disorder resulting from the process of head swivelling and crystal packing arrangements.

In most cases, the pivots described herein are in direct contact with at least one other pivot, often several others. It is unknown, however, whether these elements all function in the same time frame, as a cascade, or as separate groups of elements of an unseen grand mechanism. Mechanism can only be inferred, not proven, from examination of crystal states microstates. Intersubunit rotation may not always be correlated with head rotation or L1 stalk movement for example. Nevertheless, we suggest a two-way directional control of the head domain swivel, and intersubunit rotation that is inherent to the ribosome is mediated by EF-G. Motion induced by EF-G is envisioned to propagate from the tRNA to the intersubunit bridges, to h28 and eventually to h34 or along the same path backward.

**Figure 5.**
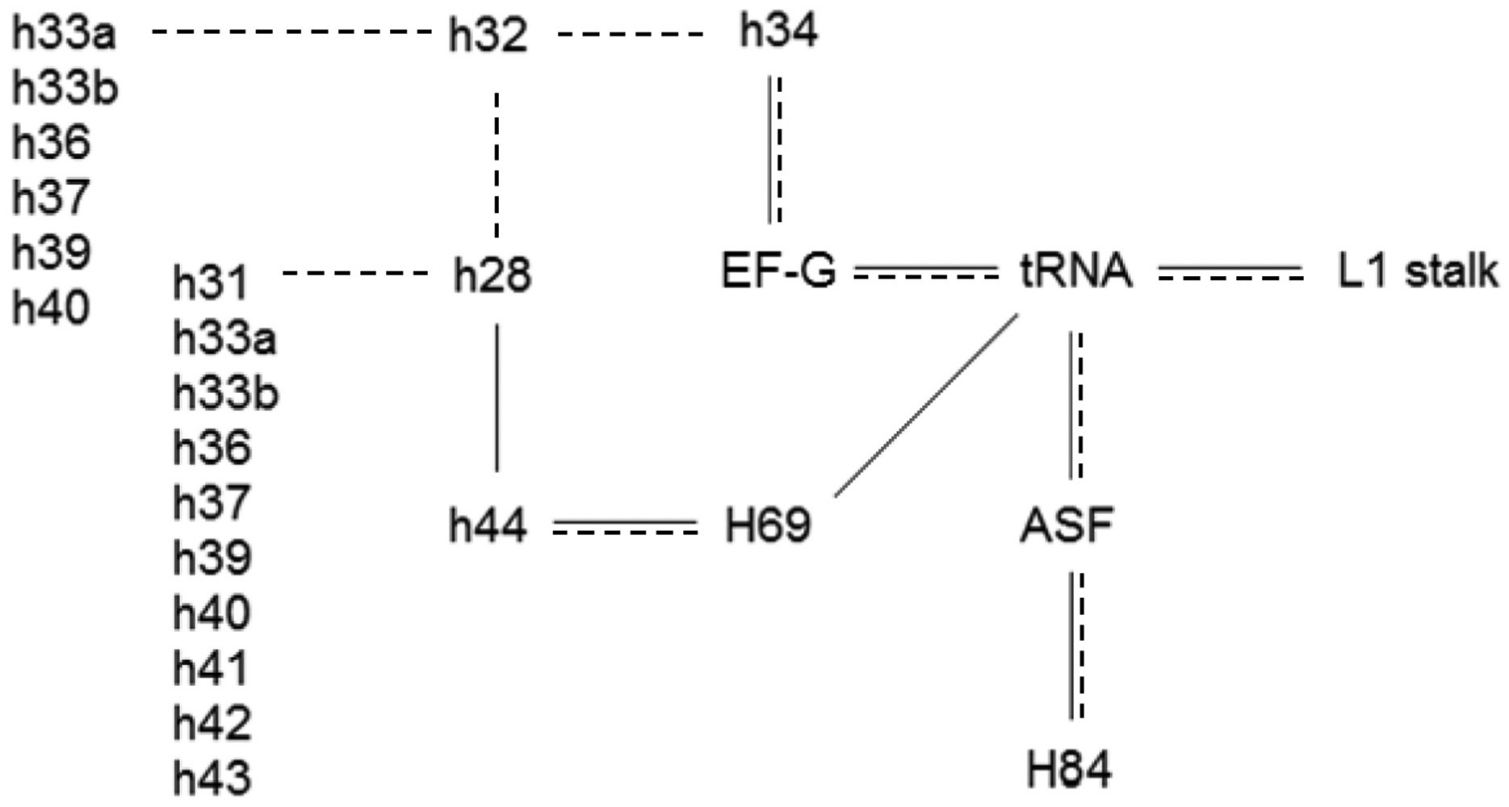
Proposed network of pivoting elements found in the ribosomal RNAs. Black lines indicate direct physical contact between moving helixes. Dashed lines indicate motion which results from an upstream pivoting motion. Helixes 28, 32 and 34 form the head domain of the SSU and lie in sequence. Helices h28 and h32 influence the motions of a number of more external pivots that are listed vertically in the figure as well as helix 34 which contacts EF-G. Thus, a cascade of motionoriginating at EF-G-GTP hydrolysis is plausible in either direction- forward towards the tRNA or in the reverse direction towards h34. Pivots which are not included on the figure are H34 which forms intersubunit bridge B4, H42 that shapes the L7/L12 stalk and h6- the spur that often docks in the P-site of the LSU allowing formation of stable crystal structures. Helices h8, h21, and h26 are poorly understood and are not included.

In the suggested network, Figure 5, the various pivot points are positioned because of their direct contact with one another, known functionality, and their dependence on the post GTP hydrolysis state of EF-G binding to the ribosome. One or more of the pivots have direct involvement in three processes—translocation, intersubunit rotation and the head swivel, including all resulting motions toward the exterior of the head domain. At the network's core is the tRNA whose structure contains a site of internal flexibility that is advantageously utilized at various times during translation (8,9) thereby allowing a series of hybrid states necessary for the translation process (21). As described recently, GTP hydrolysis results in a transition of EF-G from a compact state to an elongated form (43).

It is assumed that each EF-G analog structure compared here has taken on a similar elongated form—hence the processes described are dependent on GTP hydrolysis. The resulting domain opening induces the movement of tRNA followed by motion of the pivots in the L1 stalk and the A-site finger (44). The pivot in the L1 stalk allows it to pull spent tRNA from the P to the E site while stabilizing the P/E hybrid-state tRNA (9,15). The L1 stalk has been proposed to hinge at helix 76, which flanks a three-way junction (14). The present analysis places the pivot in helix 76 at the base pair mismatch between U2096 and G2193 which is the non-canonical pair in helix 76 that is closest to the junction. The peptidyl-tRNA elbow is rotated around the A-site finger which is especially mobile at the U871-G906 mismatch and can move by more than 10 Å from the classical to the ratcheted states (45). It is also positioned to contact the pivoting helix H84 (46).

The proposed network extends to the small subunit through the intersubunit bridges (26,47–50). H69 is associated with intersubunit bridges B2a and B2b (48,49). Helix 69 binds the tRNA at pre- and post-accommodation steps (51) as well as h44 of the SSU directly through bridge B2a (48,49,52). H69 was recently described as containing a flexible element in yeast (30). 23S rRNA helix H34 forms bridge B4 (48,49). Both H34 and H69 are known to be necessary for 70S formation (47,52). The bulge starting at position 1639 allows h44 to pivot up to 5.4 Å in order to accommodate significant intersubunit rotation and possibly information transfer. Large motions of the ribosome intersubunit rotation and the head swivel may next be connected through the h28 interaction with helixes h44 (A1394-A1500) and h32.

The observation that essentially all of the pivot points are primarily associated with non-standard pairs, bulges or occasionally three-way junctions, but not kink turns, is perhaps surprising. On the basis of molecular dynamic simulations, it has been suggested that kink turns are capable of mediating large-scale motions in the ribosome (53,54). Although there are multiple kink turns in the large rRNAs the pivoting positions reported here are not directly associated with these elements. For example, the A site finger pivot at position 871 in H42 begins earlier than the kink turn motif which starts at residue 934.

The modern ribosome is a highly evolved structure. The rRNAs are far too large to have immediately appeared in their modern form. Thus, it has been argued, as reviewed elsewhere (55), that even though large portions of the rRNA structures are largely conserved in all three domains of life, it is unlikely that even these conserved regions of the rRNAs are equally old. As a result, models are being formulated that attempt to predict the relative age of various portions of the RNA (24,56–58). All the major 23S rRNA pivot points identified here are found in RNA segments that are classified as being of similar age (24). Thus, the addition of the dynamic elements to the emerging ribosome likely occurred in a similar time frame that was relatively late in evolutionary history. This nevertheless would still be well before the last universal common ancestor.

In conclusion, for the first time, a systematic search for major pivoting elements associated with EF-G-GTP analog binding in the large ribosomal RNAs has been undertaken through the comparison of high-resolution structures. The effort provides the ribosome community with a master list of pivoting regions that can be added to when additional elements are identified or modified when locations are better refined. In addition to previously known pivot elements, multiple new pivots were identified and the previously proposed elements verified. New connectivity between elements is described including a hypothetical cascade of motions directed by EF-G. Structures from *T. thermophilus*, *E. coli* and *S. cerevisiae* show that the locations of pivots driving ribosome motion are widely conserved. This information will aid the mapping of the overall ribosome energy landscape (59) as well as rRNA–protein interactions (60). Comparison of this information to EF-TU bound structures should yield greater insight into the initiation process.

## SUPPLEMENTARY DATA

Supplementary Data are available at NAR Online.

SUPPLEMENTARY DATA
