# Supplementary material for: Major centers of motion in the large ribosomal RNAs
Source: Nucleic Acids Res. 2015 Apr 13;43(9):4640–9. doi: 10.1093/nar/gkv289 (PMC4482067; doi:10.1093/nar/gkv289)
Supplement: SUPPLEMENTARY DATA [file supp_43_9_4640__index.html]

Major centers of motion in the large ribosomal RNAs — SUPPLEMENTARY DATA 

# Major centers of motion in the large ribosomal RNAs

## SUPPLEMENTARY DATA

**Files in this Data Supplement:**

- SUPPLEMENTARY DATA
