# Supplementary material for: Major centers of motion in the large ribosomal RNAs
Source: Nucleic Acids Res. 2015 Apr 13;43(9):4640–9. doi: 10.1093/nar/gkv289 (PMC4482067; doi:10.1093/nar/gkv289)
Supplement: SUPPLEMENTARY DATA [file supp_gkv289_nar-03502-r-2014-File007.pdf]

Supplementary Information- Tables S1 to S15 and Figures S1-S5

Tables are named by organism name, followed by the names of the superimposed crystal structures, followed by which subunit was compared i.e. *E. coli* 4GD2-4KIY 16S. Tables columns are described in order as follows: the helix number which was found flexible by our method, the residues which make up the helix, the set of nucleotides comprising the stem sequence, the mismatch, bulge or three way junction (3WJ) which is believed to be the pivot, the number of angstroms the final residues move by, the RMS value generated by PyMol from superposition of the two stem elements and the number of the nucleotide the motion of which is measured. The 16S rRNA tables are further divided into 3 sections- A, B and C. Section A is the result of alignment of the stems of the external helices themselves. Section B describes motion in external pivots as a result of alignment of the stem of helix 28. Section C describes motion in external pivots as a result of alignment of the stem of helix 32.Tables S13 and S14 are the same format as main text Table 3 but instead shows measurements in *E. coli* and *S. cerevisiae*. Table S15 compares the motion potentially associated with two alternative pivots in h28.

Figure S1 shows the location of just the pivots identified in *T. thermophilus* 23S rRNA in the same manner as Figure 2 of the main text. Figures S2-S5 show the location of the pivots in the *E. coli* and *S. cerevisiae* small and large subunits in the same manner as in Figures 3 and 4 of the main text. The nomenclature is the same as in Figures 2 and 3 of the main text. However the final stems are colored in blue rather than black.

Recently the PDB has changed the way the crystal structure data is stored. Large structures containing >62 chains and/or 99999 atoms are now available on the PDB website as single "best effort" files. Previously, large structures were represented in multiple "SPLIT" entries, which have now been obsoleted. The entry name changes that are relevant to the work described here is as follows:

Thermus thermophilus

4V51 supersedes 2J00 2J01 2J02 2J03
4V5C supersedes 2WDG 2WDH 2WDI 2WDJ
4V9K supersedes 4KCY 4KCZ 4KD0 4KD2
4V9H supersedes 4JUW 4JUX
4V5F supersedes 2WRI 2WRJ 2WRK 2WRL

Escherichia coli

4V9O supersedes 4KIX 4KIY 4KIZ 4KJ0 4KJ1 4KJ2 4KJ3 4KJ4
4V9D supersedes 4GD1 4GD2 3R8S 3R8T

Saccharomyces cerevisiae

3J77 supersedes 1VXU 1VXV 1VXW
3J78 supersedes 1VXX 1VXY 1VXZ

Table S1: *T. thermophilus* 2J02-4JUW 16S rRNA

Helix	Residues	Stem	Pivot	A	RMSD	Atoms aligned	Final Residue
6	62-106	61+106	62 U-G	12.4	0.256	39	81U
8	148-174	144-147+175-178	149 Bulge	3.5	0.506	157	163C
21	588-651	586-592+647-651	593 G-U	2.3	0.392	209	619U
26	829-857	829-830+856-857	831 U-G	3.1	0.347	79	841U
44	1401-1501	1401+1501	1402 Bulge	5.4	0.293	41	1447A

A: 3’ Major Domain

31	954-1226	954-955+1225-1226	956 3WJ	0.70	0.333	76	972C
36	1068-1107	1068-1073+1102-1107	1074 3WJ	1.2	0.416	249	1078U
37	1068-1107	1068-1073+1102-1107	1074 3WJ	1	0.416	249	1092A
39	1118-1155	1118-1125+1149-1155	1125 Bulge	5.2	0.645	277	1138G
40	1113-1187	1113-1116+1184-1187	1156 3WJ	3.7	.554	163	1167A
41	1241-1296	1241-1246+1291-1296	1247 Bulge	0.50	0.487	241	1270C
42	1303-1334	1303+1334	1304 Bulge	1.60	0.346	39	1317C
43	1350-1372	1350+1372	1351 U-G	0.60	0.288	40	1362C

B: Motions resulting from h28 pivot

31	921-1396	921-922+1395-1396	1394 A	3.40	0.341	82	972C
33a	921-1396	921-922+1395-1396	1394 A	9.3	0.341	82	1030A
33b	921-1396	921-922+1395-1396	1394 A	8	0.341	82	1014A
36	921-1396	921-922+1395-1396	1394 A	3.6	0.341	82	1079A
37	921-1396	921-922+1395-1396	1394 A	1.7	0.341	82	1092A
39	921-1396	921-922+1395-1396	1394 A	4.8	0.341	82	1138G
40	921-1396	921-922+1395-1396	1394 A	2.1	0.341	82	1167A
41	921-1396	921-922+1395-1396	1394 A	6.4	0.341	82	1270C
42	921-1396	921-922+1395-1396	1394 A	6.30	0.341	82	1317C
43	921-1396	921-922+1395-1396	1394 A	4.20	0.341	82	1359

C: Motions resulting from h32 pivot

33a	984-1221	984-990+1215-1221	956 3WJ	4.70	0.438	290	1030A
33b	984-1221	984-990+1215-1221	956 3WJ	0.20	0.438	290	1014A
36	984-1221	984-990+1215-1221	956 3WJ	3.1	0.438	290	1078U
37	984-1221	984-990+1215-1221	956 3WJ	2.3	0.438	290	1092A
39	984-1221	984-990+1215-1221	956 3WJ	6.3	0.438	290	1138G
40	984-1221	984-990+1215-1221	956 3WJ	4.9	0.438	290	1167A

Table S2 *T. thermophilus* 2J01-4JUX 23S rRNA

Helix	Residues	Stem	Pivot	A	RMSD	Atoms aligned	Final Residue
34	700-732	700-702+730-732	703 Bulge	4.0	0.269	120	715G
38	868-911	911-913+868-870	871 U-G	8.3	0.32	82	884C
42	1030-1124	1030-1031+1123-1124	1032 U-G	3.1	0.369	81	1109
69	1906-1924	1906+1924	1907 G-U	4.7	0.778	38	1914C
76	2093-2196	2093-2095+2194-2196	2096 U-G	18.8	0.438	121	2168G
84	2996-2322	2996-2997+2321-2322	2298 Bulge	4.1	0.325	81	2307G

Table S3: *T. thermophilus* 2JO2- 4KDO 16S rRNA

**Helix**	**Residues**	Stem	**Pivot**	**A**	**RMSD**	**Atoms aligned**	**Final Residue**
6	62-106	61+106	62 U-G	12	0.415	35	81U
8	148-174	144-147+175-178	149 Bulge	2	0.88	165	160
21	588-651	586-592+647-651	593 G-U	2.8	0.495	201	619U
26	829-857	829-830+856-857	831 U-G	1.5	0.474	81	841U
44	1401-1501	1401+1501	1402 Bulge	13.1	0.508	42	1448C

A: 3'Major Domain

31	954-1226	954-955+1225-1226	956 3WJ	1.8	0.714	82	972C
36	1068-1107	1068-1073+1102-1107	1074 3WJ	0.9	0.631	246	1078U
37	1068-1107	1068-1073+1102-1107	1074 3WJ	2.3	0.631	246	1092A
39	1118-1155	1118-1125+1149-1155	1148 Bulge	1.6	0.863	306	1138G
40	1113-1187	1113-1116+1184-1187	1183 3WJ	5.5	0.700	166	1167A
41	1241-1296	1241-1246+1291-1296	1247 Bulge	1.2	0.717	249	1270C
42	1303-1334	1303+1334	1304 Bulge	4.7	0.428	37	1317C
43	1350-1372	1350+1372	1351 U-G	1.3	0.380	41	1359C

B: Motions resulting from h28 pivot

31	921-1396	921-922+1395-1396	1394 A	3.50	0.576	85	972C
33a	921-1396	921-922+1395-1396	1394 A	8.2	0.576	85	1030b-1028d
33b	921-1396	921-922+1395-1396	1394 A	7.5	0.576	85	1014A
36	921-1396	921-922+1395-1396	1394 A	1.4	0.576	85	1079
37	921-1396	921-922+1395-1396	1394 A	2.1	0.576	85	1092A
39	921-1396	921-922+1395-1396	1394 A	8.5	0.576	85	1137
40	921-1396	921-922+1395-1396	1394 A	2.4	0.576	85	1167A
41	921-1396	921-922+1395-1396	1394 A	6.9	0.576	85	1270C
42	921-1396	921-922+1395-1396	1394 A	6.90	0.576	85	1320
43	921-1396	921-922+1395-1396	1394 A	6.10	0.576	85	1362

C: Motions resulting from h32 pivot

33a	984-1221	984-990+1215-1221	956 3WJ	7.1	0.409	279	1014A
33b	984-1221	984-990+1215-1221	956 3WJ	5.6	0.409	279	1078
36	984-1221	984-990+1215-1221	956 3WJ	4	0.409	279	1079
37	984-1221	984-990+1215-1221	956 3WJ	1.8	0.409	279	1092A
39	984-1221	984-990+1215-1221	956 3WJ	7.1	0.409	279	1137
40	984-1221	984-990+1215-1221	956 3WJ	7.1	0.409	279	1167A

Table S4: *T. thermophilus* 2J01- 4KCZ 23S rRNA

Helix	Residues	Stem	Pivot	A	RMSD	Atoms aligned	Final Residue
34	700-732	700-702+730-732	703 Bulge	1.2	0.525	129	715G
38	868-911	911-913+868-870	871 U-G	8.9	0.499	85	883G
42	1030-1124	1030-1031+1123-1124	1032 U-G	8.1	0.536	82	1046A
69	1906-1924	1906+1924	1907 G-U	3.9	0.537	43	1914C
76	2093-2196	2093-2095+2194-2196	2096 U-G	7.2	0.537	119	2127G
84	2996-2322	2996-2997+2321-2322	2298 Bulge	3.8	0.453	80	2308G

Table S5: *T. thermophilus* 2J02-2WRI 16S rRNA

**Helix**	**Residues**	Stem	**Pivot**	**A**	**RMSD**	**Atoms aligned**	**Final Residue**
6	62-106	61+106	62 U-G	5	0.312	31	81U
8	148-174	144-147+175-178	149 Bulge	3.9	0.444	152	163C
21	588-651	586-592+647-651	593 G-U	2.3	0.377	196	619U
26	829-857	829-830+856-857	831 U-G	1	0.316	84	841U
44	1401-1501	1401+1501	1402 Bulge	6.8	0.233	39	1447A

A: 3'Major Domain

31	954-1226	954-955+1225-1226	956 3WJ	1.0	0.391	70	972C
36	1068-1107	1068-1073+1102-1107	1074 3WJ	0.9	0.373	248	1077G
37	1068-1107	1068-1073+1102-1107	1074 3WJ	0.6	0.373	248	1092A
39	1118-1155	1118-1125+1149-1155	1148 Bulge	2.8	0.610	286	1138G
40	1113-1187	1113-1116+1184-1187	1183 3WJ	3.0	0.628	165	1167A
41	1241-1296	1241-1246+1291-1296	1247 Bulge	0.3	0.444	239	1270C
42	1303-1334	1303+1334	1304 Bulge	1.9	0.283	40	1317C
43	1350-1372	1350+1372	1351 U-G	0.7	0.247	41	1362C

B: Motions resulting from h28 pivot

31	921-1396	921-922+1395-1396	1394 A	4.6	0.44	82	972C
33a	921-1396	921-922+1395-1396	1394 A	10.5	0.44	82	1028C
33b	921-1396	921-922+1395-1396	1394 A	7.5	0.44	82	1014A
36	921-1396	921-922+1395-1396	1394 A	1.2	0.44	82	1078U
37	921-1396	921-922+1395-1396	1394 A	2.1	0.44	82	1092A
39	921-1396	921-922+1395-1396	1394 A	8.7	0.44	82	1137C
40	921-1396	921-922+1395-1396	1394 A	2.4	0.44	82	1167A
41	921-1396	921-922+1395-1396	1394 A	6.9	0.44	82	1270C
42	921-1396	921-922+1395-1396	1394 A	6.9	0.44	82	1320C
43	921-1396	921-922+1395-1396	1394 A	6.1	0.44	82	1362C

C: Motions resulting from h32 pivot

33a	984-1221	984-990+1215-1221	956 3WJ	5.1	0.371	266	1030C
33b	984-1221	984-990+1215-1221	956 3WJ	0.6	0.371	266	1014A
36	984-1221	984-990+1215-1221	956 3WJ	2.3	0.371	266	1079G
37	984-1221	984-990+1215-1221	956 3WJ	1.7	0.371	266	1092A
39	984-1221	984-990+1215-1221	956 3WJ	1.5	0.371	266	1138G
40	984-1221	984-990+1215-1221	956 3WJ	3.0	0.371	266	1167A

Table S6: *T. thermophilus* 2J01-2WRJ 23S rRNA

Helix	Residues	Stem	Pivot	A	RMSD	Atoms aligned	Final Residue
34	700-732	700-702+730-732	703 Bulge	2	0.339	122	715G
38	868-911	911-913+868-870	871 U-G	7.3	0.378	86	884C
42	1030-1124	1030-1031+1123-1124	1032 U-G	2.6	0.292	79	1049C
69	1906-1924	1906+1924	1907 G-U	4.8	0.378	40	1914C
76	2093-2196	2093-2095+2194-2196	2096 U-G	9.4	0.366	116	2116G
84	2996-2322	2996-2997+2321-2322	2298 Bulge	0.9	0.325	71	2308G

Table S7: *E. coli* 4GD2-4KIY 16S rRNA

Helix	Residues	Stem	Pivot	A	RMS	Atoms aligned	Final Position
6	61-106	61+106	62 U-G	7.30	0.17	40	86G
8	144-178	144+179	145 G-G	3.50	0.299	37	160A
21	588-651	588+651	589 U-G	3.80	0.206	38	619U
26	829-857	829-831+855-857	832 G-U	2.90	0.324	117	842U
44	1401-1501	1401+1501	1402 C-A	3.50	0.239	42	1449C

A: 3'Major Domain

31	954-1226	954-955+1225A-1226	955 3WJ	0.90	0.426	80	969A
33a	997-1044	997-998+1043-1044	1042 bulge	5.80	0.675	84	1031C
33b	997-1044	997-998+1043-1044	1042 bulge	0.90	0.675	84	1014G
36	1068-1107	1068-1073+1102-1107	1074 3WJ	0.50	0.467	236	1078U
37	1068-1107	1068-1073+1102-1107	1074 3WJ	0.80	0.467	236	1092A
39	1118-1155	1118-1124+1149-1155	1125 Bulge	1.50	0.602	291	1138G
40	1117-1183	1113-1117+1183-1187	1118 3WJ	2.2	0.379	101	1167A
41	1241-1296	1241+1296	1242 G-U	0.90	0.391	38	1267C
42	1303-1334	1303+1334	1304 Bulge	3.40	0.598	43	1317C
43	1350-1372	1350+1372	1351 U-G	3.40	0.4	42	1360A

B: Motions resulting from h28 pivot

31	921-1396	921-922+1395-1396	1394 A	3.00	0.307	85	969A
33a	921-1396	921-922+1395-1396	1394 A	11.30	0.307	85	1031C
33b	921-1396	921-922+1395-1396	1394 A	9.10	0.307	85	1014G
36	921-1396	921-922+1395-1396	1394 A	0.90	0.307	85	1079G
37	921-1396	921-922+1395-1396	1394 A	1.60	0.307	85	1092A
39	921-1396	921-922+1395-1396	1394 A	incomplete	0.307	85	1138G
40	921-1396	921-922+1395-1396	1394 A	6.70	0.307	85	1168U
41	921-1396	921-922+1395-1396	1394 A	8.80	0.307	85	1267C
42	921-1396	921-922+1395-1396	1394 A	7.20	0.307	85	1317C
43	921-1396	921-922+1395-1396	1394 A	5.10	0.307	85	1360A

C: Motions resulting from h32 pivot

33a	984-1221	984C-990+1215-1221	991 3WJ	6.20	0.454	284	1031C
33b	984-1221	984C-990+1215-1221	991 3WJ	0.20	0.454	284	1014G
36	984-1221	984C-990+1215-1221	991 3WJ	2.70	0.454	284	1078U
37	984-1221	984C-990+1215-1221	991 3WJ	0.80	0.454	284	1092A
39	984-1221	984C-990+1215-1221	991 3WJ	incomplete	0.454	284	1138G
40	984-1221	984C-990+1215-1221	991 3WJ	2.10	0.454	284	1167A

Table S8: ***E. coli*** **3R8T- 4KIX 23S rRNA**

Helix	Residues	Stem	Pivot Mismatch	A	RMSD	Atoms aligned	Final Residue
34	700-732	700-702+730-732	703 U-G	2.70	0.349	123	715A
38	868-912	868-869+911-912	870 U-G	incomplete	incomplete	incomplete	incomplete
42	1030-1124	1030-1031+1123-1124	1032 U-G	7.90	0.322	81	1066U
69	1906-1924	1906+1924	1907 G-U	2.80	0.309	42	1914C
76	2093-2196	2093-2098+2191-2196	2099 U-G	incomplete	incomplete	incomplete	incomplete
84	2296-2322	2295-2296+2321-2322	2298 Bulge	1.40	0.546	62	2307G

Table S9: *E. coli* 4GD2-4GD1 16S

Helix	Residues	Stem	Pivot Mismatch	A	RMS	Atoms aligned	Final Position
6	61-106	61+106	62 U-G	7.1	0.202	43	86G
8	144-178	144+179	145 G-G	4.0	0.474	43	160A
21	588-651	588+651	589 U-G	1.9	0.448	42	619U
26	829-857	829-831+855-857	832 G-U	4.1	0.415	128	842U
44	1401-1501	1401+1501	1402 C-A	4.4	0.244	43	1450U

A: 3’ Major Domain

31	954-1226	954-955+1225A-1226	956 3WJ	0.6	0.346	80	969A
33a	997-1044	997-998+1043-1044	1042A	1.7	0.536	81	1031C
33b	997-1044	997-998+1043-1044	1042A	1.8	0.536	81	1014G
36	1068-1107	1068-1073+1102-1107	1074 3WJ	0.2	0.451	235	1078U
37	1068-1107	1068-1073+1102-1107	1074 3WJ	0.5	0.451	235	1092A
39	1118-1155	1118-1124+1149-1155	1148	0.3	0.693	287	1138G
40	1117-1183	1113-1117+1183-1187	1118 3WJ	incomplete	0.476	98	1167A
41	1241-1296	1241+1296	1242 G-U	2.2	0.468	43	1267C
42	1303-1334	1303+1334	1304 Bulge	1.8	0.683	43	1317C
43	1350-1372	1350+1372	1351 U-G	2.1	0.236	39	1360A

B: Motions resulting from h28 pivot

31	921-1396	921-922+1395-1396	1394 A	1.3	0.385	81	969A
33a	921-1396	921-922+1395-1396	1394 A	7.0	0.385	81	1031C
33b	921-1396	921-922+1395-1396	1394 A	4.2	0.385	81	1014G
36	921-1396	921-922+1395-1396	1394 A	0.6	0.385	81	1079G
37	921-1396	921-922+1395-1396	1394 A	0.5	0.385	81	1092A
39	921-1396	921-922+1395-1396	1394 A	2.4	0.385	81	1138G
40	921-1396	921-922+1395-1396	1394 A	incomplete	0.385	81	1168U
41	921-1396	921-922+1395-1396	1394 A	3.7	0.385	81	1267C
42	921-1396	921-922+1395-1396	1394 A	3.9	0.385	81	1317C
43	921-1396	921-922+1395-1396	1394 A	2.2	0.385	81	1360A

C: Motions resulting from h32 pivot

33a	984-1221	984C-990+1215-1221	991 3WJ	5.4	0.503	277	1032G
33b	984-1221	984C-990+1215-1221	991 3WJ	0.5	0.503	277	1014G
36	984-1221	984C-990+1215-1221	991 3WJ	2.2	0.503	277	1078U
37	984-1221	984C-990+1215-1221	991 3WJ	0.8	0.503	277	1092A
39	984-1221	984C-990+1215-1221	991 3WJ	1.1	0.503	277	1138G
40	984-1221	984C-990+1215-1221	991 3WJ	incomplete	0.503	277	1167A

Table S10: *E. coli* 3R8T-3R8S 23S rRNA

Helix	Residues	Stem	Pivot Mismatch	A	RMSD	Atoms aligned	Final Residue
34	700-732	700-702+730-732	703 U-G	7.20	0.214	112	715A
38	868-912	868-869+911-912	870 U-G	incomplete	incomplete	incomplete	incomplete
42	1030-1124	1030-1031+1123-1124	1032 A-G	2.90	0.264	70	1067A
69	1906-1924	1906+1924	1907 G-U	3.50	0.447	37	1914C
76	2093-2196	2093-2098+2191-2196	2099 U-G	17.00	0.866	247	2145C
84	2296-2322	2295-2296+2321-2322	2297 A-G	2.6	0.631	62	2307G

Table S11: ***S. cerevisiae*** **1VXW-1VXZ 18S rRNA**

Helix	Residues	Stem	Pivot Mismatch	A	RMS	Atoms aligned	Final Residue
6	57-90	57+90	58 U-G	3.4	0.144	34	75U
8	139-175	139-142+172-175	143 Bulge	1.8	0.665	168	159U
21	744-807	744-745+806-807	746 Bulge	11.9	0.728	77	781U
26	1039-1079	1039-1040+1078-1079	1042 Bulge	4.8	0.577	78	1059U
44	1638-1764	1638+1764	1639 Bulge	18.1	0.089	36	1699G

A: 3' Major Domain

31	1179-1459	1179-1180+1458-1459	1181 3WJ	3.3	0.328	83	1193A
33a	1037-1224	1220-1224+1259-1263	1225 Bulge	3.5	0.578	204	1241G
36	1288-1327	1288-1290+1325-1327	1291 G-G	3.8	0.816	124	1298U
37	1288-1327	1288-1290+1325-1327	1291 G-G	4.2	0.816	124	1309C
39	1338-1386	1338-1345+1380-1385	1346 Bulge	4.6	0.569	262	1363U
40	1333-1418	1333-1337+1414-1418	1387 3WJ	7.1	0.647	180	1399C
41	1471-1538	1471+1538	1481 Bulge	2.9	0.667	290	1503A
42	1540-1571	1540+1571	1541 G-A	1.7	0.409	42	1553G
43	1587-1611	1587+1611	1587 A-U	2.2	0.949	40	1597A

B: Motions resulting from h28 pivot

31	1145-1633	1145-1146+1632-1633	1631 A	0.90	0.290	74	1193A
33a	1145-1633	1145-1146+1632-1633	1631 A	5.1	0.290	74	1241G
36	1145-1633	1145-1146+1632-1633	1631 A	0.9	0.290	74	1298U
37	1145-1633	1145-1146+1632-1633	1631 A	3	0.290	74	1309C
39	1145-1633	1145-1146+1632-1633	1631 A	5.8	0.290	74	1363U
40	1145-1633	1145-1146+1632-1633	1631 A	3.7	0.290	74	1399C
41	1145-1633	1145-1146+1632-1633	1631 A	2.8	0.290	74	1503A
42	1145-1633	1145-1146+1632-1633	1631 A	5.90	0.290	74	1553G
43	1145-1633	1145-1146+1632-1633	1631 A	1.80	0.290	74	1597A

C: Motions resulting from h32 pivot

33a	1209-1454	1209-1212+1451-1454	1213 3WJ	0.7	0.601	168	1241G
36	1209-1454	1209-1212+1451-1454	1213 3WJ	8.3	0.601	168	1298U
37	1209-1454	1209-1212+1451-1454	1213 3WJ	6.6	0.601	168	1309C
39	1209-1454	1209-1212+1451-1454	1213 3WJ	6.6	0.601	168	1363U
40	1209-1454	1209-1212+1451-1454	1213 3WJ	7.4	0.601	168	1399C

Table S12: *S. cerevisiae* 1VXU- 1VXX 26S rRNA

Helix	Residues	Stem	Pivot Mismatch	A	RMSD	Atoms aligned	Final Residue
34	831-863	831+863	832 G-U	3.4	0.393	43	846A
38	985-1064	985+1064	986 U-G	5.2	0.301	31	1024G
42	1205-1299	1205-1207+1297-1299	1208 U	4.3	0.447	119	1259A
69	2249-2267	2249+2267	2250 G-U	2.9	0.158	31	2257C
76	2435-2512	2435-2436+2511-2512	2437 G-U	8.6	0.438	79	2472U
84	2665-2691	2665-2666+2690-2691	2667 bulge	2.3	0.312	76	2676A

Table S13: 4GD2-4KIY *E. coli* 16S rRNA

Helix	Helix 28	Helix 32	Individual
**31**	3.00	**NA**	0.60
**33a**	11.30	6.20	5.80
**33b**	9.10	0.20	0.90
**36**	0.90	2.70	0.60
**37**	1.60	0.80	0.80
**39**	incomplete	incomplete	incomplete
**40**	6.70	2.10	2.2
**41**	8.80	**NA**	0.90
**42**	7.20	**NA**	3.40
**43**	5.10	**NA**	3.40

Table S14: *S. cerevisiae* 1VXW-1VXZ 18S rRNA

Helix	Helix 28	Helix 32	Individual
**31**	0.90	NA	3.3
**33a**	5.1	0.7	3.5
**36**	0.9	8.3	3.8
**37**	3	6.6	4.2
**39**	5.8	6.6	4.6
**40**	3.7	7.4	7.1
**41**	2.8	NA	2.9
**42**	5.90	NA	1.7
**43**	1.80	NA	2.2

Table S15. Motions resulting from two potential pivot points in h28 in *T. Thermopilus*

B: Motions resulting from 1394A bulge

31	921-1396	921-922+1395-1396	1394 A	3.40	0.341	82	972C
33a	921-1396	921-922+1395-1396	1394 A	9.3	0.341	82	1030A
33b	921-1396	921-922+1395-1396	1394 A	8	0.341	82	1014A
36	921-1396	921-922+1395-1396	1394 A	3.6	0.341	82	1079A
37	921-1396	921-922+1395-1396	1394 A	1.7	0.341	82	1092A
39	921-1396	921-922+1395-1396	1394 A	4.8	0.341	82	1138G
40	921-1396	921-922+1395-1396	1394 A	2.1	0.341	82	1167A
41	921-1396	921-922+1395-1396	1394 A	6.4	0.341	82	1270C
42	921-1396	921-922+1395-1396	1394 A	6.30	0.341	82	1317C
43	921-1396	921-922+1395-1396	1394 A	4.20	0.341	82	1359

D: Motions resulting from 926G bulge

31	921-1396	921-924+1392-1396	1391 Bulge	0.7	0.324	168	972C
33a	921-1396	921-924+1392-1396	1391 Bulge	**5.3**	0.324	168	1030G
33b	921-1396	921-924+1392-1396	1391 Bulge	2.8	0.324	168	1014A
36	921-1396	921-924+1392-1396	1391 Bulge	1.0	0.324	168	1078U
37	921-1396	921-924+1392-1396	1391 Bulge	0.9	0.324	168	1092A
39	921-1396	921-924+1392-1396	1391 Bulge	**8.0**	0.324	168	1138G
40	921-1396	921-924+1392-1396	1391 Bulge	2.0	0.324	168	1167A
41	921-1396	921-924+1392-1396	1391 Bulge	1.5	0.324	168	1270C
42	921-1396	921-924+1392-1396	1391 Bulge	1.7	0.324	168	1317C
43	921-1396	921-924+1392-1396	1391 Bulge	1.0	0.324	168	1359

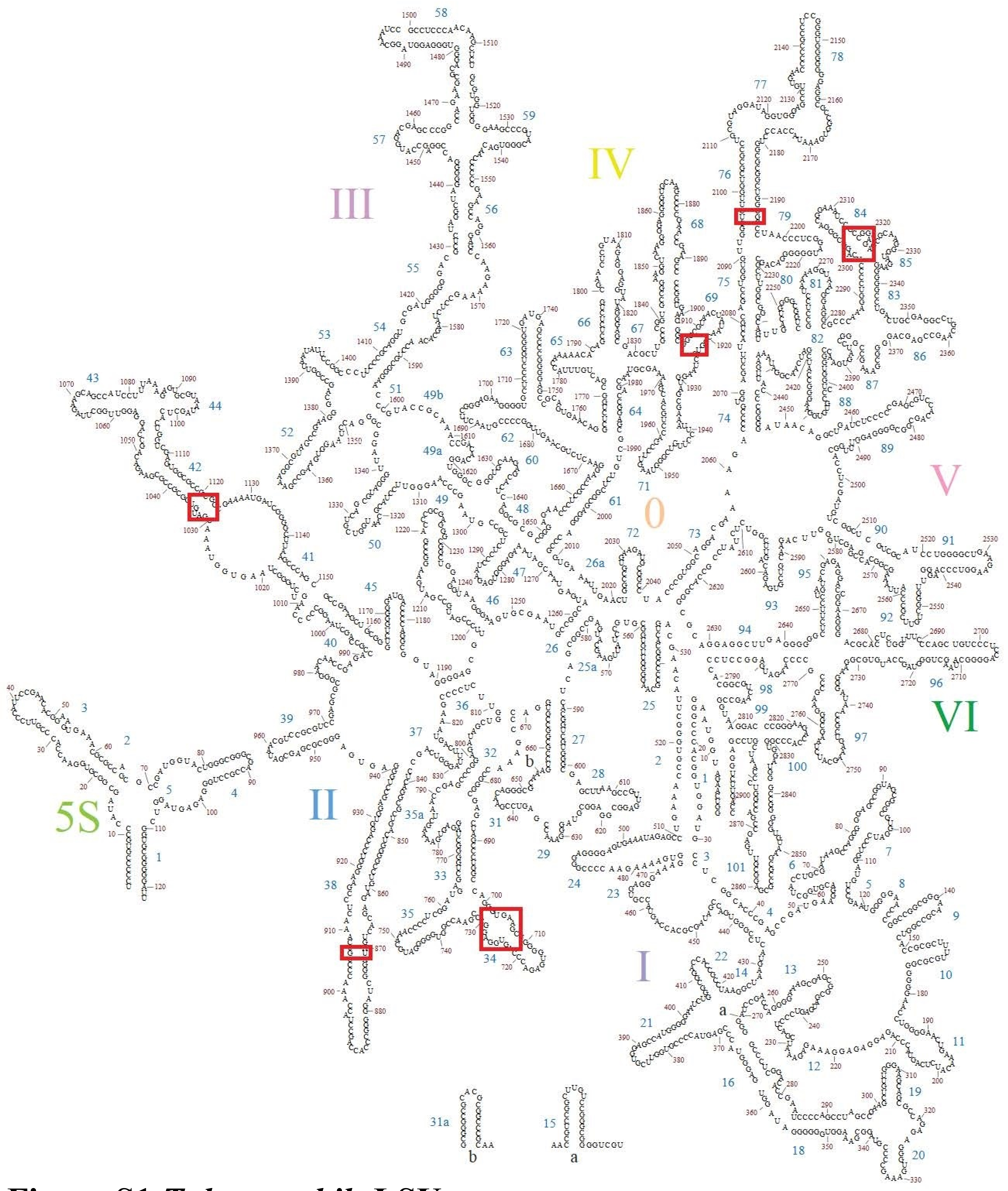

Figure S1 ***T.thermophils*** **LSU**

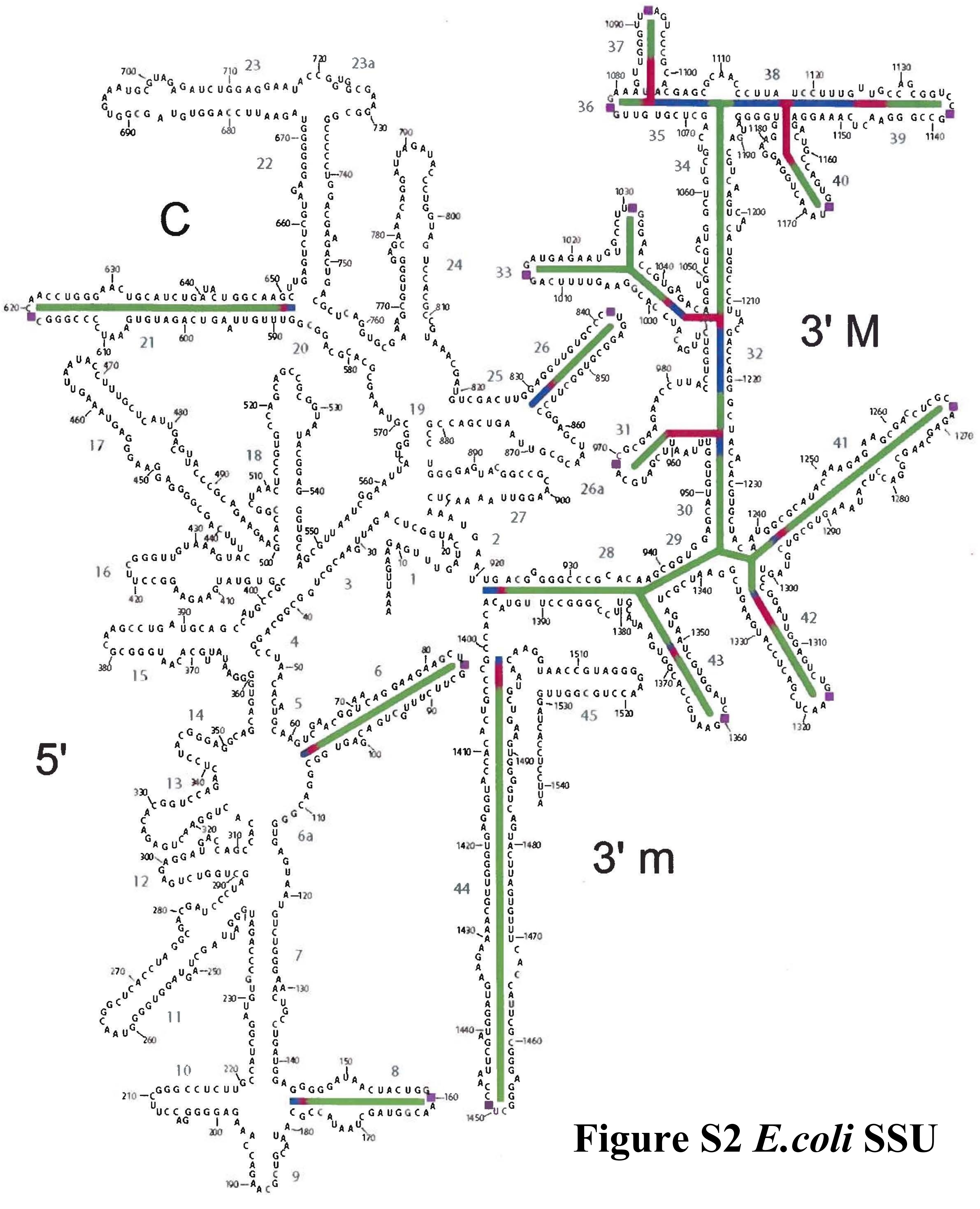

Figure S2 *E.coli* SSU

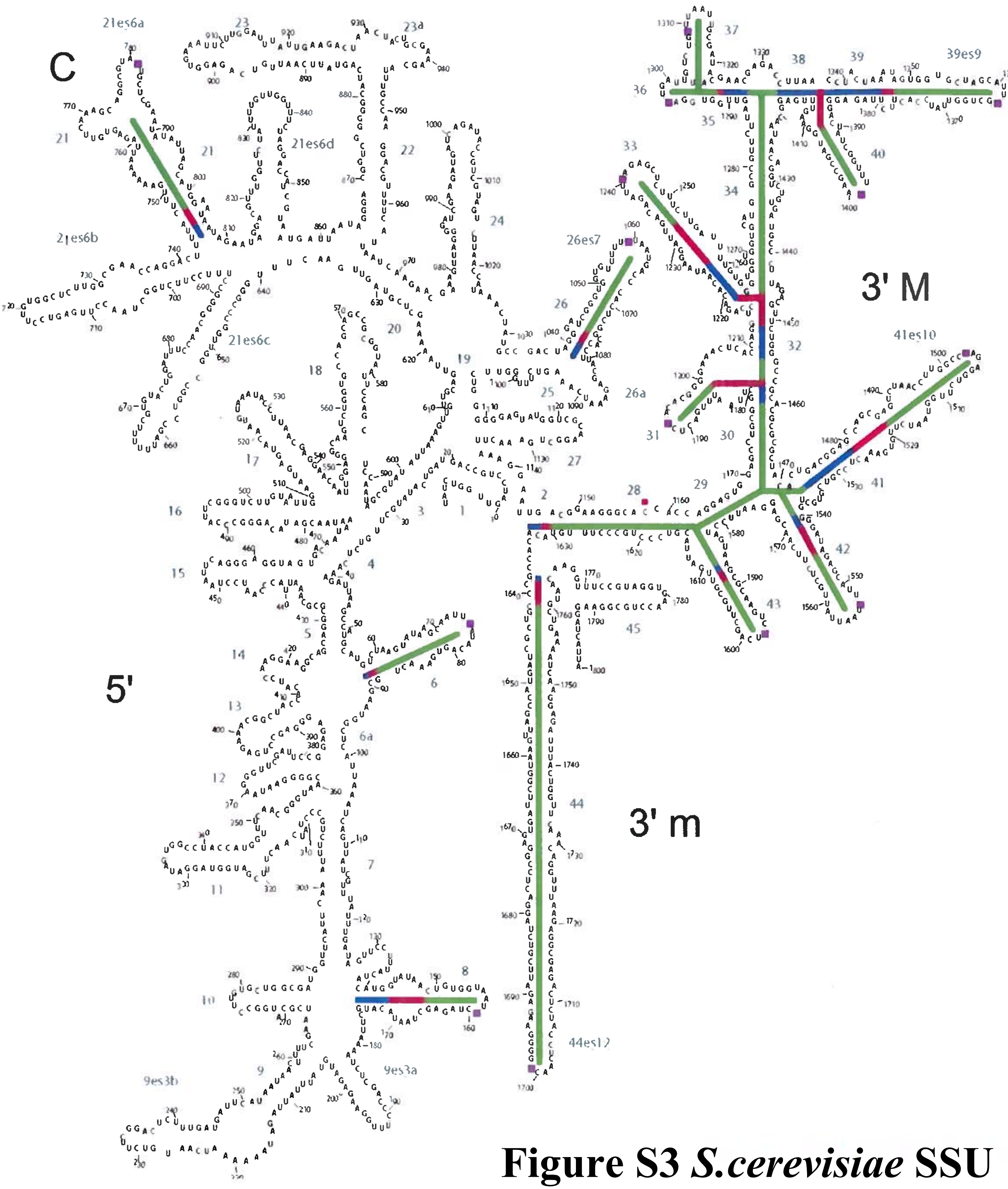

Figure S3 ***S.cerevisiae*** **SSU**

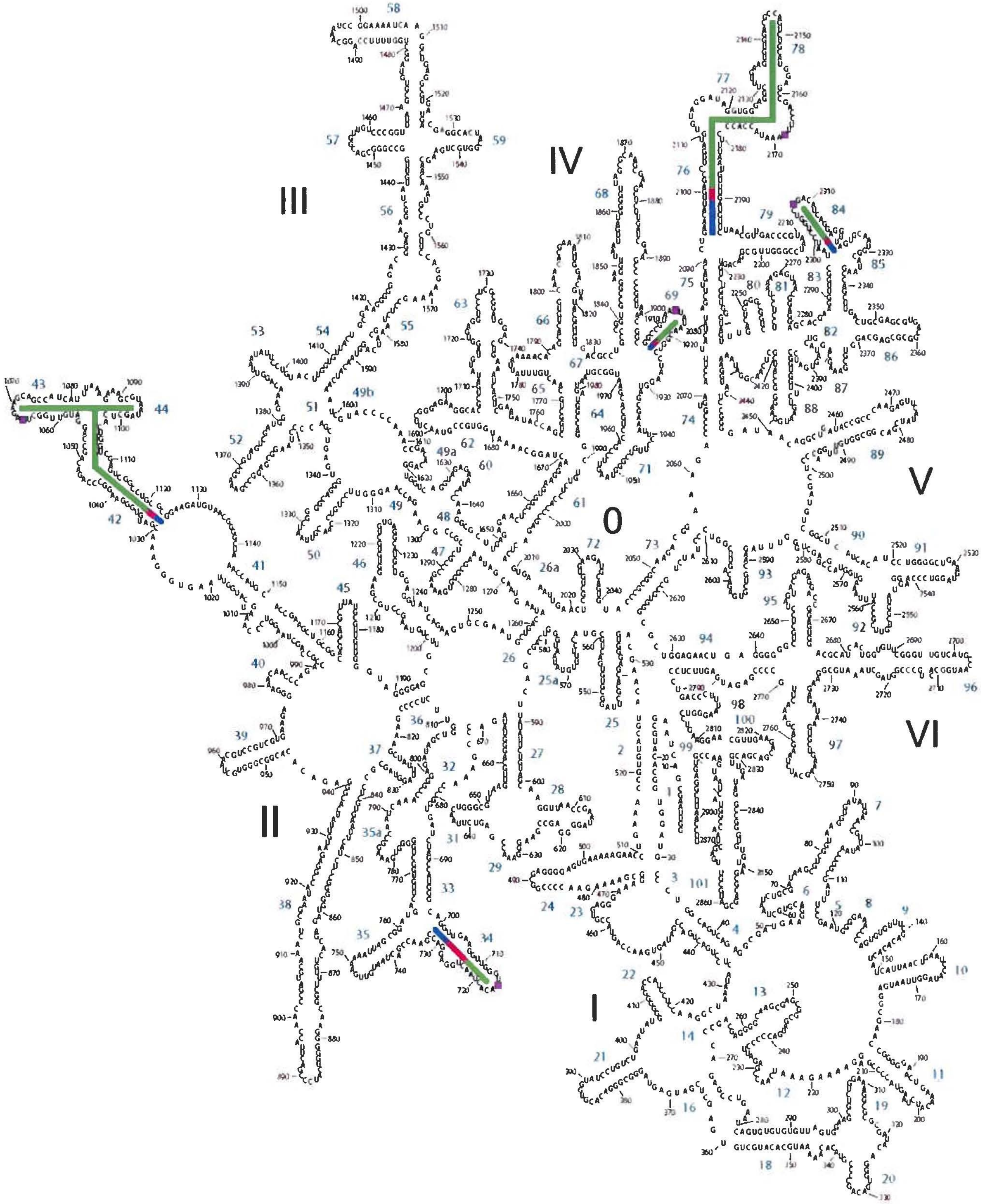

Figure S4 *E.coli* LSU

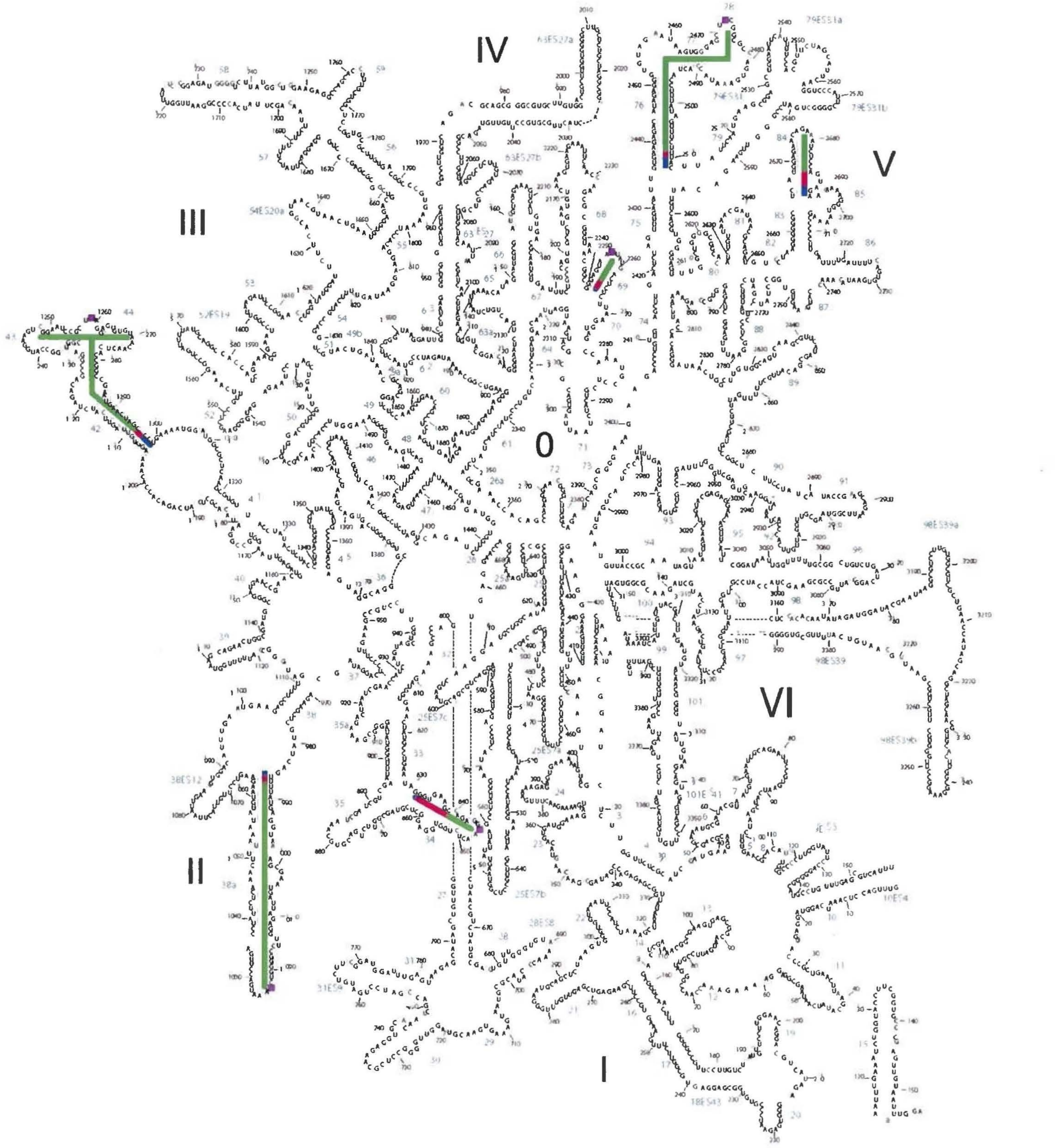

Figure S5 *S.cerevisiae* LSU
